# Pain Management Strategies in Intensive Care Unit: Challenges and Best Practice

**DOI:** 10.31661/gmj.v13i.3264

**Published:** 2024-04-29

**Authors:** Habib Zakeri, Pantea Mahtosh, Mohammad Radmehr, Roohollah Rahbani, Leala Montazeri, Saba Moalemi, Parisa Mahdiyar, Farnaz Hemati, Aliasghar Karimi

**Affiliations:** ^1^ Research Center for Neuromodulation and Pain, NAB Pain Clinic, Shiraz University of Medical Sciences, Shiraz, Iran; ^2^ Kaiser Permanente Santa Clara Medical Center, Homestead Campus, Santa Clara, USA; ^3^ I.M. Sechenov First Moscow State Medical University, Moscow, Russia

**Keywords:** Intensive Care Unit, Pain, Opioid, NSAID, Anesthesia

## Abstract

Pain management in the ICU (intensive care unit) is a very complex problem which involves a wide variety of conditions, lack of sufficient tools for use, and high personnel to patient ratio. In the last three decades, pain as a clinical issue has become well analyzed, and treatment protocols based on scientific evidence have been established. Besides medication, some non-pharmacological methods such as music therapy, relaxation, and massage have been proven to be very much practical and manageable in pain management of ICU. The main opioids are utilized predominantly due to their power but NSAIDs and local anesthesia are combined with opioids with the aim to reduce the pain as much as possible. Yet more research now has to prove that pain evaluation and management is effective. This article discusses on the issues and the best approaches to solving them when managing pain in ICU patients.

## Introduction

ICU patients are at a high risk to suffer pain due to the complications of surgery or invasive procedures which, in particular, have procedures like burns, cancer, critical illness, and surgeries as their people [1][2][3].

Also, the sympathetic activation and hypermetabolic stress response are common in pain which lead downwards and deplete body proteins as thus, blood glucose levels are elevated, immunosuppression occurs, the wound healing is impaired, the mineral levels are altered, and inflammatory markers are increased. Hence, the particularization and the resolution of pain during the stay of patients in the intensive care unit (ICU) are the most important issues for improving patients’ outcomes [3][4][5][6].

Nevertheless, till now, the very locality of the experienced pain is rather unclear and the people usually complain about pain when they are shaving, washing themselves, pressing themselves to the back part of the body, changing sheets, and repositioning themselves.

The role of monitoring the pain is becoming highly predictive for the successful treatment outcomes, thus many valid instruments for pain measurement are being developed. Such instruments may consist of numeric scales, pain scales that involve behavior, and devices to registered nociception as well as pain accompanied by the trained specialists [4][7][8][9].

The selection of pain management strategy in intensive care unit (ICU) patients ought to encompass both pharmacological and nonpharmacological approaches. It is indispensable to carry on regular pain assessments with adequate interventions before opening the door to sedative drugs. Moreover, there is the need for standardized techniques, including the use of accepted methods, in order to conduct a thorough evaluation in light of the complex nature of pain in these patients. As pumping out the rules and regulations for pain management in intensive care unit is essential to guarantee a smooth approach to this problem [3][4][10][11].

This paper reviewed the problems and the strategies for pain management in ICU patients.

## Non-pharmacological Methods

Non-pharmacological (non-drug) approaches to pain management in the ICU may be opioid-sparing and analgesia-enhancing, available at a fraction of the price, easy to provide and safe [5][11][12].

However, there are few studies that have reported the outcomes of applying needle therapy as an alternative to drugs in pain patients [11][12][13].

Relaxation Rechniques

The non-pharmacotherapy methods which include deep breathing, imagery, progressive muscle relaxation, and meditation are of low-cost, usage-less, and safe as prompt pain management in ICU patients’ treatment [14].

The slowness and pseudo coherent of deep breathing is what makes it an effective tension reducer and serene muscle tension elasticity. This involves of muscle contractions and relaxation of various muscle groups, to achieve muscle tension reduction and pain relief [1][14][15]. However, this is not applicable if a patient has severe respiratory distress or cardiovascular instability [2][15][16].

Music Therapy

In addition, the music therapy had been proven to have numerous positive effects on the patients, especially with the intensive care unit ones. It is known to achieve relaxation, decrease anxiety, and trimming pain. Researches showed that the patients with ICUs take so much load back in pain with their half hours of music therapy which is enough to cut and reduce pain [17][18].

Besides music therapy has likely supported the communication process between patients’ families, too [19].

Some studies have demonstrated that the patients who received live music therapy have experienced the pronounced decreasing of heart rate, breathing rate and agitation levels 7 years ago 6 years older [17][20]. Music therapist should be cultivating treatment relevance by applying individual choices and cultural aspects of the patient when selecting music. A therapist should achieve this by designing and playing music that patients can connect to the healing process. The positive result of this is that the therapy intervention will be more effective [21].

## Other Non-pharmacological Interventions

Picture-guided spiritual care: Spirituality of a person is not just following a particular religion and a few practices. This method uses visual aids so that the patient can clearly see their spiritual needs and preferences. The study conducted by Berning et al. has shown that this method is full of success as it has decreased the anxiety levels and improved the spiritual support of adult ICU patients on mechanical ventilation [22][23].

Cognitive-behavioral: Both pain and anxiety experienced by patients can be effectively addressed by specialized nursing protocols and non-pharmacological techniques as multiple studies have shown [24][25].

Acupuncture: Acupuncture therapy has been found to play a major role in both preoperative and intensive care areas. Pain and nausea among patients can be significantly reduced, as well as the consumption of narcotic medication [26][27].

Virtual Reality (VR) and hypnosis: Talking about hypnosis, which is a conventional medical procedure, brings the patient into a focused and trance-like state in which he becomes highly receptive to suggestions that are given to him. In the opposite direction, VR technology should be applied in an environment that the patient will like, maybe the beach, etc. with the use of three-dimensional device and may contribute to such reduction of pain. These interventions are known to be very helpful in terms of post anesthesia pain, anxiety and stress because of the reduction of their scores in these categories in a number of recent studies. [28][29]

Massage therapy: Beauty increases are reported to lessen anxiety and decrease postoperative pain among patients in ICU [30][31][32]. Furthermore, foot massage is an effective way to reduce the pain [32][33].

Moreover, combination of massage and music therapy has proven effective than just using both of them on its own: it does not only relief the pain, but also improves the vital signs in ICU patients [34].

Various strategies have been investigated in ICU patients to enhance spiritual care, address pain, and alleviate anxiety [22][23][24][25][26][27][28][29][30][31][32][33][34].

One intervention that can be used is spiritual care guided by pictures, in which patients use images to recognize their spiritual needs and preferences [22][23]. Berning at al. demonstrated that this method is viable and successful in decreasing anxiety and enhancing spiritual support in adult ICU patients on mechanical ventilation [22]. Moreover, cognitive-behavioral methods like cognitive restructuring, relaxation, and distraction techniques have been shown to effectively reduce pain and anxiety in ICU patients [24][25].

Acupuncture is now seen as a feasible and positively received alternative care choice in the ICU, offering potential advantages in decreasing pain, nausea, vomiting, and the use of narcotics [26][27].

Nevertheless, more studies are required to determine its effectiveness in alleviating pain. The use of virtual reality (VR) and hypnosis have demonstrated potential in lessening pain, anxiety, and stress in ICU patients [28][29].

furthermore, several studies suggested that massage therapy, such as foot massage and massage therapy with music, can enhance sleep quality, alleviate pain, and enhance vital signs for patients in the ICU [30][31][32][33][34].

In general, these interventions provide potential ways to enhance the well-being of ICU patients, but more research and assessment are necessary to fully comprehend their effectiveness and influence.

Ultimately, the aforementioned interventions provide various ways to possibly impact the quality of life for patients that would likely help them cope with the challenges of being hospitalized in the ICU, notably the need for more research and evaluation to wholly understand their efficacy and power.

## Pharmacological Treatments

**Table:** Table1. The Common Drugs, Mechanism of Action, and Side effects to Control Pain in Critically Ill Patients [42][43].

**Drug Category**	**Mechanism of Action**	**Side Effects**
Opioids	binding to opioid receptors in the brain, spinal cord, and other areas of the body, reducing the perception of pain.	respiratory depression, sedation, constipation, nausea, vomiting, tolerance, and dependence.
Nonsteroidal Anti-inflammatory Drugs (NSAIDs)	inhibit the production of prostaglandins, which are chemicals that promote inflammation, fever, and pain.	gastrointestinal ulcers and bleeding, kidney damage, increased risk of cardiovascular events (such as heart attack or stroke), and fluid retention.
Acetaminophen (Paracetamol)	inhibiting the production of prostaglandins in the brain.	liver damage (especially in cases of overdose), allergic reactions, and skin rash.
Gabapentinoids (Gabapentin, Pregabalin)	modulating the activity of certain neurotransmitters involved in the transmission of pain signals.	dizziness, drowsiness, peripheral edema, weight gain, and ataxia.
Ketamine	NMDA receptor antagonist and provides analgesic effects by blocking the transmission of pain signals.	hallucinations, dissociation, elevated blood pressure, tachycardia, and respiratory depression.
Local Anesthetics (e.g., Lidocaine)	signals in a specific area, providing temporary pain relief.	local tissue irritation, allergic reactions, and systemic toxicity if absorbed in large amounts.
Alpha-2 Agonists (e.g., Dexmedetomidine)	sedative and analgesic effects by activating alpha-2 adrenergic receptors in the brainstem.	bradycardia, hypotension, dry mouth, and sedation.
Corticosteroids (e.g., Methylprednisolone)	- anti-inflammatory properties and may be used to reduce inflammation and pain associated with certain conditions.	immunosuppression, hyperglycemia, fluid retention, mood changes, and osteoporosis.

Within the ICU, it is a particularly important to achieve a stable tradeoff between effectively treating pain in critically ill patients and minimizing the side effects that could be as a result of treatment. As seen in Table-1, there are many different medications and their side effects that are used in controlling pain amongst critical patients. A perfect pharmacological regimen should be put in place to keep this balance [35][36][37][38][39][40][41][42][43].

Analgesic Medications

Pain management is achieved by using opioids as the oldest and strongest drug in the intensive care unit (ICU). Probably, the most well-known opioids are morphine sulfate, fentanyl, and sufentanil (pain relievers). The opioids specific are the most effective than the other painkillers medication for pain management. Nevertheless, opioids are susceptible to accumulation and poisoning in patients with renal failure, so they should be used carefully.

The most common opioids’ side effects are such as nausea, vomiting, and constipation, with some of them fading after a long-term morphine use (Table-1).

A comprehensive list of taking all the prescription drugs should be shared with the healthcare team to rule out drug interactions and side effects [41][42][43].

However, NSAIDs are restricted for pain management for ICU patients because of the dangers of GI bleed, platelet dysfunction, or renal failure.

But in the event that NSAIDs are used by low-risk patients who are also not on the other nephrotoxic medications, they reduce opioid use and the severity of the pain experienced by these patients. On the other hand, the effect of NSAIDs on the ICU admission and duration of ventilator use is still undefined [44].

Acetaminophen is a painkiller that raises the pain threshold and inhibits the ascent of pain pathways. This medication might be very potent in decreasing the grade of pain and the intake of opioids. But paracetamol is not better than opioids for pain control [45][46][47][48][49][50].

Ketamine can provide patients with dissociative sedation and analgesia on the ICU who are on mechanical ventilation. It may be regarded as an additional treatment for pain, agitation, and delirium when it is given in low dose. Ketamine may be useful in the critically ill setting but administering it requires careful attention and frequent monitoring for any side effects [51][52][53][54].

Local Anesthetics

Different local anesthetic methods to administer exist and they include the continuous wound infiltration and the topical spraying or cream of the agent [55][56]. A variety of factors influences the effectiveness of local anesthetics in the pain management for the different structures. Nonetheless, according to the investigations, regional and systemic anaesthetics are safe and the adverse effects are rarely observed with appropriate dosage, for example, hypotension and respiratory depression [57][58].

Multimodal Analgesia

This is the strategy with more frequency in the ICU units and healthcare facilities are encouraged to devise pain management strategies including multimodal analgesia [37][59][60]. Recognizing the potential of non-opioid analgesics, such as Non-Steroidal Anti-Inflammatory Drugs, in conjunction with use of low-dose opioids when applicable is foundational. Providing an intervention with multimodal analgesia to reduce pain in critically ill adult patients, the mortality, ventilation time utilizing machines, and infection rates will be minimized through its opioid reduction effect. Patient centered pain algorithms rely simultaneously on physician training and development, nursing, physical therapy and pharmacy services [60][61].

Multimodal analgesia has been linked to reduction in death rate, length of mechanical ventilation clock, and infection, a lot of which has been established by decreasing opioid intake. It was found that patients on multimodal analgesia had higher likelihood of number of organ failures and hypnotics than patients on opioids only. The use of alternatives to opioids like NSAID’s alongside low-dose opioids where ever practical is a great step in reducing the dependency on opioids. this method enables the pain relief and minimizes organ dysfunction so as this would overall improve patient outcomes [37][59][60][61].

Nerve Blocks

The nerve block is one of the pain management modalities in which local anesthetics or other medicines are given with a needle to the specific nerve which then blocks the pain signals from reaching the brain. Nerve blocks could help to relieve pain and even lead to the complete loss of sensations if is needed for surgery. Neuraxial analgesia and peripheral nerve blocks are techniques often used to combat pain based on internal disease or accident in the critically ill adult patients [36][62][63][64]. The higher number of patients trying out the locoregional analgesia techniques like nerve blocks implemented in the ICU is also happening. Nonetheless, nerve blocks have hazards and some specifications, and their use should be thoughtfully assessed with every unique situation by an anesthesiologist [63][65][66]. The possible complications of nerve blocks are nerve injury, catheter infection, bruising and toxicity. For example, the surgical contraindications that would not be suitable in ICU patients are systemic sepsis, vasopressor therapy, and coagulopathy. Considering the scarcity of hard evidence, utmost care should be well observed when performing nerve blocks in ICU and monitoring for possible complications [66][67][68].

## Challenges

Though the pain management has been developed highly, the pain in the ICU of the patients is the complicated issue which is still going on. One of the key drawbacks is absence of precise protocol for management of pain in the ICU [43][69][70].

Although there are established principles for pain management in general, they could be ineffective when applied to the custom needs of ICU patients [43]. Besides, people with opioid addiction and tolerance makes a hard task for surgeons, because usual opioid pain medication may not work or harm their health [71].

The pain management process can be burdened by the obsolete clinical practices and unproductive systems and this needs the continuous education and improved pain management protocols Pain in critically ill patients may be underestimated due to the fact that even after many years of research and development of guidelines, such pain is still often untreated [72][73].

This might be a result of the many different factors, such as the complication of pain measurement in patients with the mental state of reduced consciousness or paying attention to the fleeting pain that is provoked by procedures [74].

Concerns hereby have led to the development of multimodal pain management as the most efficient method to manage ICU pain. This methodology merges non-drug options (for example, music therapy and relaxation methods) with analgesic drugs to offer a multi-dimensional and personalized approach to pain alleviation [72].

Although the application of multimodal analgesia in the ICU setting is not an easy task, research shows that it can be made possible [74]. ICU nurses should be accustomed to validated methods of pain assessment and have a standardized protocol for the whole multidisciplinary team on the way they assess, document, and communicate patients` pain [43]. Thus, there has to be an ongoing education and training to make sure that all the team members are provided with the right resources and skills to better manage pain in ICU patients. Along with these issues, there is a noticeable need for the additional research on the consequences of pain management in the ICU [73][74].

While non-pharmacological approaches have shown promise, there is still a lack of evidence on their effectiveness and optimal use in this setting. As such, it is crucial for healthcare professionals to continue to explore and evaluate different approaches to pain management in the ICU, in order to provide the best possible care for patients [26].

## Conclusion

The basic strategy of pain management in the ICU patients are multimodal analgesic in which the patient receives more than one analgesic agent or pharmacological agents with different action mechanism to guarantee reduced side effects while increased pain control [74]. Beside the diagnostic and therapeutic tools respectively, injectable opioids as the first-line of pain medication are also prescribed by some health professionals. Pharmacological intervention should always be avoided and non-pharmacological methods should be chosen. In addition to pain assessment and prevention, pain reassessment and prevention too should be formulated in pain management strategies [74].

From the basic physical and psychological pain, a patient experiences to adequately administer pain management and relieve pain [75]. Each healthcare institution is answerable for the identification of its very own set of regulations for pain management in ICU units. Nonetheless, medicine is one of the professions which has rules considered both required and essential. This guideline Hinders the undesirable effects of pain interventions as well as optimizes analgesic efficacy for patients in ICU [74].

Though the evolution in pain management techniques has proved helpful in managing pain in the patients admitted in the ICU, it is clear that there are still some challenges that need to be tackled head on. Through the multimodal analgesia and always trying to improve pain management routines, healthcare professionals can be on their way to give the patients in the unconscious state effective and appropriate pain control. Nevertheless, the process of research as well as education needs to be developed and perfected to be able to see the complications of pain management for this group of patients and to continue improving the treatment of critically ill patients. Additional trials are required to demonstrate which strategy maybe the most beneficial for pain assessment and treatment in patients who are in intensive care.

## Conflict of Interest

There is no conflict of interest to be declared regarding the manuscript.
